# Supplementary material for: Associations between defensive victim-blaming responses (DARVO), rape myth acceptance, and sexual harassment
Source: PLoS One. 2024 Dec 4;19(12):e0313642. doi: 10.1371/journal.pone.0313642 (PMC11616802; doi:10.1371/journal.pone.0313642)
Supplement: Attachment — Submitted filename: Response to Reviewers.docx [file pone.0313642.s001.docx]

**Response to reviewer comments**

PONE-D-23-33892

11 July, 2024

We would like to thank the reviewers for providing thorough and attentive feedback on our original manuscript. We found the comments to be very helpful in strengthening the quality of our manuscript during revisions.

We have responded to the reviewer comments below. Original reviewer comments are italicized, and our responses appear below each in red text.

*Reviewer #1: This manuscript presents an exploration of DARVO techniques on sexual harassment, sexual perpetration, and rape myths in MTurk and student samples. Results indicated that there were significant associations between DARVO and the outcomes doe both samples. I commend the authors for their work on this manuscript, but there are a few things that need to be addressed before I would consider it for publication:*

*1. When discussing rape myth, I think it needs to be clearer that the literature you cite seems to be mainly focused on women as victims. There are a different set of rape myths for men, and we don’t know very much about the rape myths for people outside of the binary. I think this distinction could be more clear in this section of the paper. This should also be made clear in the methods as well.*

We have made changes in both the literature review and the method section to clarify the type of rape myths featured in our manuscript. The section introducing rape myths in the literature review includes specific gendered language and now states:

“Most research has focused on rape myths in the context of male-perpetrated sexual violence against women [Suarez & Gadalla, 2010]. The notion that women compel male perpetrators to commit sexual offenses through their actions or appearance is a common rape myth, as are the beliefs that women lie about being assaulted and that men unintentionally commit sexual assault by way of intoxication or uncontrollable sexual desire. Decades of research on rape myths about male perpetrators and female victims have produced stable findings…”

In the method section, the following clarification was added to the description of the study’s measure of rape myth acceptance: “This measure contains 22 items representing four categories of harmful beliefs about male-perpetrated sexual violence against women.”

*2. Having demographics tables may help with readability and interpretability of these sections. It is a lot of information to keep in mind and it would be easier to have a table to look back at for quick reference.*

We have compiled the demographics information for both the undergraduate and community sample into a table (Table 1). Consequently, we have also simplified the written description of the demographics in the participants section.

*3. Were there any questions assessing if the DARVO techniques were used in a sexual harassment/victimization scenario? If not, I think it needs to be clear in the abstract (and maybe even title) that you’re looking at DARVO broadly and not necessarily in a sexual harassment/victimization scenario. I was surprised to find it was a general use of these items when I got this this section as it is currently written. I think this needs to be more clear in the methods and discussion sections as well.*

We have made several changes in accordance with this comment. In the abstract, we added a sentence noting, “We examined whether people who use DARVO as a means of responding to confrontations involving a range of wrongdoings also engage in behaviors and ascribe to beliefs that contribute to sexual violence.

The current study section now specifies this general use of DARVO as well (new additions highlighted in yellow): “The purpose of the current study was to evaluate individuals’ own general use of DARVO as a response to confrontations regarding a range of wrongdoing, including possible incidents of sexual violence as well as a variety of other incidents, and their rape myth acceptance. To further explore DARVO’s connection to sexual violence, the current study also sought to investigate the general use of this tactic in conjunction with a behavioral measure of engagement in sexual harassment.”

The materials section describing the DARVO measure has been revised to include the following information:

“For the current study, modifications were made so that the scale evaluated respondents’ own general DARVO use during a time they were confronted by someone else. Respondents were instructed to think of “the worst or most serious thing” they were accused of doing and were then asked report on this experience. The scale did not impose limitations with respect to when the confrontation occurred or the specific nature of the confrontation – respondents could report on a confrontation that happened at any time and, as long as it was about the worst or most serious thing they were accused of doing, could be about a range of behaviors. In this way, DARVO use was assessed across a variety of potential contexts.”

The first paragraph of the discussion section has been revised to provide more clarity about the way DARVO use was evaluated in the current study (see highlighted text below):

“The purpose of the current study was to explore associations between general DARVO use in diverse confrontational contexts, rape myth acceptance, and sexual harassment perpetration in undergraduate and community samples. In support of hypotheses, both undergraduates and community adults who use DARVO as a response to being confronted over a range of wrongdoings reported greater acceptance of rape myths (H1a and H1b) and were more likely to perpetrate sexual harassment (H2a and H2b). Otherwise stated, individuals who issue denials, personal attacks, and reversals of victim and offender roles as a means of responding to confrontations are more likely to hold victim-blaming beliefs and sexually harass others. These findings suggest that general DARVO use indicates greater support for and participation in rape culture.”

*4. Why was the DoD version of the SEQ selected? That version was originally designed to capture these behaviours in the military. It seems like an odd choice for use at a civilian university and with a community sample. More information is needed.*

The 16-item DoD version of the SEQ (SEQ-DoD-s), published by Stark and colleagues (2002), was selected primarily for its psychometric properties. Stark et al. (2002) employed item response theory to pare down and standardize the SEQ, which resulted in a shortened sexual harassment questionnaire with good psychometric qualities and a four-factor structure. And, although it seems unusual to use a sexual harassment scale originally intended to be used among military populations, the measure’s items are worded in general terms and apply to a variety of contexts. Moreover, Stark et al. (2002) note that the SEQ-DoD is commonly used among civilian populations.

The following brief sentence was added to the manuscript’s description of the SEQ-DoD-s: The “SEQ-DoD-s was selected due to its relatively limited number of items and for its good psychometric properties [32].”

*5. What were the other measures and where did these fall within the order that they were presented to participants?*

The other measures in the full survey were related to personality constructs (e.g., the big five, dark triad traits). We have clarified the nature of these other measures and provided information regarding its position in relation to the main study variables:

“All participants were presented with the study measures in the same order (i.e., demographic questions, DARVO-USE, rape myth acceptance, and sexual harassment perpetration) within a larger survey containing personality measures unrelated to the current study’s hypotheses. The personality measures appeared prior to the DARVO-USE items.”

*6. Your discussion is currently quite short and not a fulsome discussion of the current results. I would suggest organizing it by hypothesis and providing more detail throughout. I think it needs to be very clear that the DARVO measure here is not necessarily reflective of sexual violence. The way it is currently written seems to assume that or brush over the fact that these could be fairly mundane confrontations described by participants. Their behaviours to these confrontations may not necessarily be the same as if they were confronted with abuse, harassment, or sexual violence. This seems like it would probably be the case, but it is an untested assumption. Also, although it was not the main goal of the study, the differences in demographics bears mentioning in the discussion. You have compared two samples and found there to be different patterns, that can’t simply be brushed to the side and not discussed. This section could do with some expansion overall.*

We have reorganized the discussion section to better reflect the order of hypotheses. Moreover, we have expanded the discussion throughout. The newly added text is highlighted in yellow in the discussion section.

We understand that the unrestricted way we measured DARVO allowed respondents to report on a range of different experiences that could range from trivial to severe. In light of this, we have added the following to our discussion of study limitations:

“Further, the measure prompts respondents to report on a confrontation about the worst or most serious thing they were accused of doing, but it does not limit what the “worst or most serious thing” should be. Respondents were therefore able to report on a range of experiences, including some experiences which might have been relatively trivial. This is a potential limitation of the current study. Given the findings from the current study identify a connection between DARVO use and both rape myth acceptance and sexual harassment perpetration, future research would benefit from exploring DARVO use specifically among people who have been confronted about sexual misconduct.”

In response to comments from the second reviewer, we have included additional analyses with demographic variables in this letter. We also mention the possible role of demographic differences in our discussion section:

“This difference [in correlation strength between the two samples], while notable, is not central to our hypotheses; however, it suggests differences in some demographic variables may attenuate the strength of the associations between DARVO, rape myth acceptance, and sexual harassment perpetration. Additional DARVO research that focuses specifically on the role of education level, socioeconomic status, and other demographic variables would be better equipped to explore this issue.”

One reason we have chosen to not explore demographic variables in more depth is that the pattern of findings is the same with the exception of associations with the Deny subscale (i.e., the community sample resulted in significant associations between the Deny subscale and RMA/sexual harassment, and the undergraduate sample did not). If we had found more discrepant findings, then an expanded exploration of demographic differences would potentially be elucidating. However, we believe our current study indicates relatively stable findings across two samples of differing characteristics.

*7. You are also potentially missing out on the voices of trans and non-binary individuals in your sample, which is also an important limitation.*

We thank this reviewer for drawing attention to diverse gender identities with respect to our sample. We have noted this limitation in our discussion section:

“Additionally, the number of nonbinary and trans individuals was relatively small in both samples. Future research that probes DARVO and its connection to rape culture would benefit from sampling larger proportions of non-white and gender diverse individuals to better represent the racial and gendered makeup of people living in the US.”

*Reviewer #2: Thank you for the opportunity to review your work. Generally speaking, understanding the factors that contribute to sexual harassment, or any form of sexual violence, is important. This study examines the relationships between several different factors, including DARVO, rape myth acceptance, and sexual harassment perpetration and compared findings across two sample sources. While there are some strengths, I also believe there are areas where the study/manuscript could be improved. Specifically, the conceptual overlap between rape myths and DARVO should be further explored and untangled in the literature review, elements of the methodology need clarification, and the limitations section should be expanded. Below, I detail my recommendations in the general order of the paper’s outline.

Introduction

Please be more specific about the prevalence of sexual violence against male victims. “Less than a third” could be anywhere between 0 and 33%, making it not very meaningful.*
The language has been updated to be more precise. It now reads, “More than half of women and approximately 31% of men living in the US report experiencing sexual violence at some point during their lives.”

*The introduction paragraph/section needs a “so what” paragraph or some paragraph connecting the issue of sexual violence to the research questions posed in the current study, as well as why this study is important.*

We have added the following paragraph to the beginning of the Current Study section of the introduction:

“Both rape myths and DARVO threaten the wellbeing of sexual violence victims and promote victim-blaming narratives. Moreover, DARVO is an interpersonal behavioral tactic that undermines victims’ credibility by portraying victims of sexual violence as dishonest, unreliable, or vindictive. When sociocultural environments support narratives that deny victimization and cast blame on victims, perpetrators are less likely to face consequences for their harmful behaviors and are potentially more likely to continue committing abuses. In this way, perpetrators’ use of DARVO may help sustain sexual violence by contributing to a victim-blaming culture and by cultivating social conditions that allow for continued perpetration. Exploring DARVO’s connection with attitudes known to contribute to sexual violence (i.e., rape myths) as well as to sexual violence perpetration is critical for understanding this tactic’s role in rape culture.”

*Given the use of sexual harassment as a variable in analyses, it would make more sense to focus discussion of the problem of sexual violence on this specific type of sexual violence. I expect more people have both experienced sexual harassment and perpetrated it, compared to rape or sexual assault. So describing the frequency/prevalence of sexual harassment could make the problem this paper addresses seem more important, pervasive, and relevant.*
We have included additional information about sexual harassment (i.e., definitions, prevalence) to the introduction section. The following paragraph was added:

“Sexual harassment is a prevalent type of sexual violence that is experienced across a variety of contexts. Although definitions vary [10], sexual harassment typically includes unwanted sexual advances (verbal or physical), requests for sexual favors, and gender harassment (e.g., derogatory or offensive remarks about gender) [11]. In the US, one in three women and one in nine men report experiencing sexual harassment in a public space [1]. Within institutions of higher education, this rate is higher: approximately 75% of transgender, genderqueer, or gender nonconforming undergraduates, 62% of cisgender female undergraduates, and 43% of cisgender male undergraduates enrolled in colleges and universities have been targets of sexual harassment [12]. As with other forms of sexual violence, sexual harassment is associated with a number of adverse consequences, including psychological distress, depression, and alcohol misuse [13].”

*Literature review

After reading the literature review, I was unsure what the conceptual difference was between DARVO and rape myths. The authors state on page 6 that DARVO “echoes” rape myths and even offers an example of how, so I am confused how the DARVO responses are different from just communicating rape myth beliefs in a specific situation? It might be helpful to include some of the specific DARVO statements that relate to each component. These are listed in the methods section description of this variable, but including examples of both DARVO and rape myths sooner could help clarify. Also, please clarify if DARVO is used in response to any type of perpetration or confrontation, or is specific to sexual violence-related perpetration and confrontations. In reading this section, because the authors focus on DARVO among sexual offenders, it sounded like this behavioral response was specific to sex offenders/sexual violence. If that is the case, then I am not sure there really is much difference between DARVO and rape myth acceptance. DARVO would simply be the statements that transform rape myths—which are essentially neutralizing definitions—into explicit statements. For example, if a person believes the accidental rape myth, or that men rape accidentally when they are too turned on or intoxicated, then they may say DARVO related statements, like, “I didn’t mean to do,” “It was an accident,” “You should not have worn that dress/made out with me if you didn’t want to have sex,” or “You should have said no more clearly.” My point is that DARVO responses to a sexual violence situation seem likely the result of rape myth acceptance. If the concept of DARVO was developed and studied primarily in the area of sexual violence offending, then I am not sure there is sufficient conceptual difference between DARVO and rape myths. So, unpacking this and clarifying these concepts and their differences is essential.*

We appreciate this discussion about DARVO and rape myths, and we welcome the opportunity to distinguish the two concepts both here and in the manuscript.

In brief, DARVO is a behavioral process that occurs interpersonally and can be deployed in a wide range of contexts, including (and, according to its conceptual underpinnings, especially) as a means of deflecting accountability when individuals engage in sexual misconduct. In other words, DARVO is a defensive procedure that seeks to protect reputations, escape blame, and avoid possible consequences. It functions as a mechanism of personal defense. In comparison, rape myths are a set of false beliefs about sexual violence that are rooted in patriarchy; these beliefs are culturally prevalent and typically blame victims of sexual violence and excuse actions of perpetrators.

Rape myths are beliefs that people accept or reject to varying degrees. On the other hand, DARVO is something that people *do*. While both rape myths and DARVO can lead to undesirable outcomes like victim blaming, they are expressed differently (behavioral vs. attitudinal) and have different functions (self-protection vs. cultural support of patriarchal violence).

When DARVO is used by someone who has committed sexual violence, then the content of their DARVO responses may echo common rape myths; for example, the perpetrator may assert his victim is making a false accusation of rape to intentionally cause him harm. This reflects a common rape myth (i.e., that women lie about being raped), but does not encompass all rape myths, nor does it necessarily represent the perpetrator’s own acceptance of rape myths.

Moreover, while our current study has identified statistically significant associations between DARVO and rape myths, these associations are not so strong as to suggest that these concepts are indistinguishable.

The following paragraph was added to the end of the DARVO section of the introduction:

“Despite the parallels between DARVO responses and rape myths, these are conceptually distinct concepts. DARVO is an interpersonal behavioral response used as a mechanism of self-defense; it primarily functions to deflect accountability. Rape myths, on the other hand, are widely held cultural beliefs that are rooted in patriarchal violence. An individual using DARVO to evade blame for sexual violence may invoke certain rape myths in the process of using the self-defense tactic, but DARVO does not encompass societal – or even personal – beliefs about sexual violence.”

*Methods*

*I am confused by the confrontation component of the survey (. I am generally very confused by how this component was assessed. I understand how the DARVO behaviors/responses were measured. That is not my concern. My issue is with the confrontation they reflected on when responding to the DARVO questions. Were they exposed to a hypothetical confrontation event and asked how they would respond? Or were they asked to recall a confrontation that they experienced? If this second option, were they told to recall any confrontation or only a confrontation related to sexual harassment? If any confrontation, did the authors control for the nature of the confrontation? I expect that DARVO use might vary depending on if the confrontation was about sexual violence, some other crime, or some less concerning conflict, like who left the lights on in an empty house or some such mundane issue. Also, was there a timeframe for the confrontation, such as, within the last week, last month, or last year? Who was doing the confronting? Was it the victim or some third party? Given the confrontation is what precipitated the DARVO responses, clarifying what this was and how it was measured would be helpful.*
We have clarified the DARVO use measure in the materials section of the revised manuscript. Specifically, we added:

“Respondents were instructed to think of “the worst or most serious thing” they were accused of doing and were then asked report on this experience. The scale did not impose limitations with respect to when the confrontation occurred, with whom the confrontation occurred, or the specific nature of the confrontation – respondents could report on a confrontation that happened at any time and, as long as it was about the worst or most serious thing they were accused of doing, could be about a range of behaviors. In this way, DARVO use was assessed across a variety of contexts.”

Acknowledging this may be a limitation of the current study, we also added the following to the discussion section:

“Further, the measure prompts respondents to report on a confrontation about the worst or most serious thing they were accused of doing, but it does not limit what the “worst or most serious thing” should be. Respondents were therefore able to report on a range of experiences, including some experiences which might have been relatively trivial. This is a potential limitation of the current study. Given the findings from the current study identify a connection between DARVO use and both rape myth acceptance and sexual harassment perpetration, future research would benefit from exploring DARVO use specifically among people who have been confronted about sexual misconduct.”

We agree that DARVO use may vary depending on the incident that provoked the confrontation. We think it is possible that individuals who use DARVO to argue against more mundane accusations would also be likely to use DARVO for more serious incidents as well.

*Later the authors explain that some cases involved individuals who were innocent, and some involved people who were guilty of whatever action they were confronted about. Then it sounded like they included both groups because the diagnostic tests showed no difference between their responses or the relationship between relevant variables. This is a problem. There are very different reasons why a person who is innocent would use DARVO and why a person who is guilty would use DARVO. The innocent person would reasonable deny their perpetration because they actually didn’t do anything wrong. They also would reasonably claim that they were being victimized by the accusations. If not DARVO, then how else might innocent people respond to confrontation/accusations? My point is that, there is no good theoretical reason that DARVO responses to a confrontation about something you did not do would be indicative of rape myth acceptance or sexual harassment perpetration. So even if statistically, the relationships between these variables are the same in all the groups, the reasons for these relationships should be different, and so the groups should be analyzed separate. I would argue the nonsignificant difference between the groups indicates a conceptual problem with the study’s framework. What should differentiate people who use DARVO because they didn’t do something versus those who use DARVO when they did do something? I understand why RMA might predict DARVO use among the guilty, but why would it predict DARVO use among the innocent? Even the guilty may not believe they are guilty if they truly believe rape myths. That is, a person who believes victims deserve rape if they make out with you first may not believe they perpetrated are rape if foreplay was involved.*

We appreciate the thoughtful engagement and comments on this topic. DARVO, even when used as a means of defense against demonstrably false allegations (i.e., DARVO use among the innocent), is still an antisocial response. It is entirely reasonable, as this comment points out, for individuals to deny a truly false allegation; this by itself is typically not problematic. However, the two remaining elements of DARVO – attack and reverse victim and offender – are more harmful ways of defending oneself. We imagine, for example, that a university dean accused of sexual misconduct by a student would categorically deny the allegations – this would be expected and unsurprising. But if the dean were to personally attack the student by, for instance, suggesting the student is mentally ill or has ulterior motives, then the dean’s self-defense adopts a new dimension beyond denial that would potentially make a restorative and positive outcome more difficult to achieve. Reversals of victim and offender could further contribute to a social environment on campus that is not conducive to productive resolutions.

There are other ways people who are falsely accused of wrongdoing may respond that do not involve DARVO. Like stated above, issuing denials without engaging in social combat (i.e., attack and reverse victim and offender) is one such way. People who are falsely accused may also express confusion, curiosity, concern, or the desire to seek a resolution. None of these responses necessitate attacking the accuser or using reversals of victim and offender. As such, the ARVO aspect of DARVO is a pernicious and largely unnecessary part of defending oneself.

Because of this, we argue that DARVO is a harmful way of responding to confrontations, even when the person being confronted believes themself to be innocent. The inherently antisocial and aggressive nature of DARVO, regardless of the context of its use, could help explain why DARVO use among innocent respondents would also be associated with rape myth acceptance and sexual harassment.

Individuals’ perception of their innocence in the current study is another point of discussion. It is possible that some people may genuinely believe in (and therefore report on) their own innocence, even if they are truly guilty of what they are accused. The odds of this inaccurate self-categorization may increase with DARVO use, too. In other words, those who were more likely to use DARVO responses during a confrontation may also be more likely to wrongly believe they are innocent. As the current study was not able to verify whether participants were innocent or guilty in the experiences they reported on, the Innocent/Guilty distinction may not be legitimate for all participants.

This comment serves as a good reminder that there are many pathways for future DARVO research to explore. For the current study, however, we believe that it is acceptable to examine DARVO use in both contexts to evaluate this tactic’s associations with rape myths and sexual harassment.

*Given the sample is sufficiently large to look at only the group who responded to the confrontation about something they did do, I recommend only using that group and excluding the other group (the innocents).*

We appreciate this suggestion for an alternative approach to our analyses. However, given the response we have provided above, we think it is appropriate to include both DARVO use contexts in the present study.

Briefly, however, we ran correlations separated by Innocent/Guilty groups for both samples. Results for the undergraduate sample (Table A) and community sample (Table B) are displayed below. Correlations for the Innocent confrontation context are shown below the diagonal. Correlations for the Guilty confrontation context are shown above the diagonal.

| **Table A.**  *Bivariate Correlations for Study Variables among Participants in the Undergraduate Sample (N = 602), Split by Innocent/Guilty Confrontation Context.* | | | | | | |
| --- | --- | --- | --- | --- | --- | --- |
|  | 1. | 2. | 3. | 4. | 5. | 6. |
| 1. DARVO total | -- | .693*** | .854*** | .852*** | .140* | .123* |
| 2. Deny | .599*** | -- | .493*** | .323*** | .019 | .077 |
| 3. Attack | .833*** | .289*** | -- | .611*** | .131* | .060 |
| 4. Reverse | .865*** | .318*** | .591*** | -- | .153** | .140* |
| 5. Rape myth acceptance | .164** | .087 | .175** | .128** | -- | .299*** |
| 6. Sexual harassment perpetration | .143** | .008 | .178** | .129* | .273*** | -- |
| ***p* < .01. ****p* < .001. Correlations for the Innocent confrontation context are shown below the diagonal. Correlations for the Guilty confrontation context are shown above the diagonal. | | | | | | |

| **Table B.**  *Bivariate Correlations for Study Variables among Participants in the Community Sample (N = 335), Split by Innocent/Guilty Confrontation Context.* | | | | | | |
| --- | --- | --- | --- | --- | --- | --- |
|  | 1. | 2. | 3. | 4. | 5. | 6. |
| 1. DARVO total | -- | .811*** | .955*** | .937*** | .595*** | .602*** |
| 2. Deny | .625*** | -- | .727*** | .660*** | .542*** | .560*** |
| 3. Attack | .942*** | .432*** | -- | .822*** | .551*** | .603*** |
| 4. Reverse | .946*** | .544*** | .820*** | -- | .551*** | .504*** |
| 5. Rape myth acceptance | .587** | .214** | .598*** | .563*** | -- | .643*** |
| 6. Sexual harassment perpetration | .681** | .357*** | .658*** | .652**** | .662*** | -- |
| ***p* < .01. ****p* < .001. Correlations for the Innocent confrontation context are shown below the diagonal. Correlations for the Guilty confrontation context are shown above the diagonal. | | | | | | |

When separated by Innocent/Guilty confrontation context, the associations between study variables do not tend to show different patterns. This, in conjunction with other results finding that controlling for Innocent/Guilty confrontation contexts do not alter the main findings of the multivariate analyses (see below), suggests there might not be a statistical need to only present data from the Guilty confrontation context. Moreover, we would prefer to present our analyses with as much statistical power as possible; this would mean retaining our full sample.

*The authors say they used “Multiple Linear Regression” to estimate the effects of RMA and sexual harassment perpetration on DARVO. My understanding of MLR is that it is a general term describe a linear regression that uses multiple predictors and a single outcome. But, there are different ways to estimate these models, such as using OLS or HLM. Can the authors clarify the specific type of regression/test they performed?*

We used General Linear Model (GLM) procedures to conduct a multivariate multiple regression in SPSS, which uses OLS estimation. We have clarified this in the results section. Moreover, we have updated the name of the test we used throughout the manuscript; originally, we referred to it as a “multiple linear regression,” but “multivariate multiple regression” is ultimately more accurate as it describes multiple independent variables and multiple dependent variables.

*The models presented in table 3 and 6 are hard to read. Typically, predictors for each outcome/model are grouped together, and then model level statistics like the Wald X2, log likelihood, and/or R2 are included to show model fit. As presented, it looks like the authors ran 6 models, 3 (one for each DARVO component) using RMA as a predictor and 3 (one for each DARVO component) using sexual harassment perpetration as a predictor. If that is the case, then this is not “multiple” linear regression, as there is only one predictor. You might consider reorganizing the table following the example below:*


*Variable Outcome 1*
*B SE t P CI*
*V1*
*V2*
*V3*
*Metric1 - - -*
*Metric 2 - - -*
*Outcome 2*
*V1*
*V2*
*V3*
*Metric1 - - -*
*Metric 2 - - -*
*Outcome 3*
*V1*
*V2*
*V3*
*Metric1 - - -*
*Metric 2 - - -*

As we used a General Linear Model (clarified above and in the revised manuscript) to conduct a multiple multivariate regression, this analysis does have multiple simultaneous predictors (i.e., sexual harassment perpetration and RMA). This is different from running multiple individual regression models with single predictor variables.

We have reformatted the two tables (now Table 6 and Table 7) to increase clarity. Table 6 appears below as an example of the revised table format.

| **Table 6**. **Regression Coefficients for a Multivariate Multiple Regression Analysis Predicting Deny, Attack, and Reverse Subscales from Rape Myth Acceptance and Sexual Harassment Perpetration in the Undergraduate Sample (*N* = 602).** | | | | | |
| --- | --- | --- | --- | --- | --- |
|  | Deny | | | | |
| **Predictor variables** | *B* | *SE* | *t* | *p* | 95% CI |
| Rape myth acceptance | .059 | .095 | .620 | .536 | [-.127, .245] |
| Sexual harassment perpetration | .144 | .206 | .701 | .484 | [-.260, .548] |
|  | Attack | | | | |
|  | *B* | *SE* | *t* | *p* | 95% CI |
| Rape myth acceptance | .100 | .035 | 2.87 | .004 | [.031, .168] |
| Sexual harassment perpetration | .161 | .075 | 2.14 | .033 | [.013, .168] |
|  | Reverse | | | | |
|  | *B* | *SE* | *t* | *p* | 95% CI |
| Rape myth acceptance | .099 | .041 | 2.39 | .017 | [.017, .180] |
| Sexual harassment perpetration | .218 | .090 | 2.43 | .015 | [.042, .394] |

*Did the authors control for any of the participants’ demographic characteristics or if the innocent/guilty confrontation condition? If not, they should in the MLR models.*

In the method section, we described how participants reported their DARVO use in two different contexts: innocent and guilty. In this description, we included a link that provided study analyses that controlled for innocent/guilty confrontations, which did not meaningfully differ from the analyses we originally presented in the manuscript. Our description in the method section states:

“In the current study, which uses data originally collected for multiple projects, participants were randomly assigned to report their DARVO use in one of two contexts: during a confrontation over a wrongdoing they committed, or during a confrontation over a wrongdoing they did not commit. This contextual element was included during data collection for the purposes of a separate study and is not part of the current study’s hypotheses regarding individuals’ general DARVO use. However, to ensure that asking participants to report DARVO use in the two different contexts did not significantly impact the study’s findings related to rape myth acceptance and sexual harassment perpetration, correlations and multivariate analyses were computed while both controlling and not controlling for this effect. This set of analyses (controlling for and not controlling for DARVO use contexts) did not substantially differ in their findings for either the undergraduate or community samples. Correlations and multivariate analyses are therefore presented without the contextual variable for the sake of simplicity and clarity. Results for the analyses that control for this variable have been made available [here](https://osf.io/fny7z/?view_only=128890beedda4bc4bc88c9463cafe067).”

We had not originally included demographic variables in our multivariate models as the models were primarily exploratory and we had no known theoretical or empirical basis for including demographic variables as controls. Moreover, the one demographic variable we were interested, participant gender, was not associated with differences in DARVO use in either of the samples.

We have re-run the multivariate multiple linear regression using GLM controlling for demographic variables and for confrontation type. Given the lack of racial diversity in our samples, we elected to not include racial identity as a control variable. Table C (undergraduate sample) and Table D (community sample) below show the results of these analyses.

| **Table C**.  *Regression Coefficients for a Multivariate Multiple Regression Analysis Predicting Deny, Attack, and Reverse Subscales from Rape Myth Acceptance and Sexual Harassment Perpetration in the Undergraduate Sample (N = 566).* | | | | | |
| --- | --- | --- | --- | --- | --- |
|  | Deny | | | | |
| **Predictor variables** | *B* | *SE* | *t* | *p* | 95% CI |
| Rape myth acceptance | .151 | .102 | 1.48 | .139 | [-.049, .350] |
| Sexual harassment perpetration | .118 | .209 | .565 | .572 | [-.292, .528] |
| Age | -.015 | .017 | -.923 | .356 | [-.048, .017] |
| Religiosity | .111 | .042 | 2.62 | .009 | [.027, ,194] |
| Political orientation | .081 | .038 | 2.13 | .033 | [.006, .156] |
| Gender | -.093 | .110 | -.847 | .397 | [-.308, .122] |
| Confrontation type | .433 | .073 | 5.90 | < .001 | [.289, .577] |
|  | Attack | | | | |
|  | *B* | *SE* | *t* | *p* | 95% CI |
| Rape myth acceptance | .117 | .039 | 3.03 | .003 | [.041, .193] |
| Sexual harassment perpetration | .199 | .080 | 2.50 | .013 | [.042, .355] |
| Age | -.008 | .006 | -1.25 | .213 | [-.020, .005] |
| Religiosity | .034 | .016 | 2.09 | .037 | [.002, .065] |
| Political orientation | .000 | .014 | .018 | .986 | [-.028, .029] |
| Gender | -.130 | .042 | -3.12 | .002 | [-.212, -.048] |
| Confrontation type | .025 | .028 | .877 | .381 | [-.030, .079] |
|  | Reverse | | | | |
|  | *B* | *SE* | *t* | *p* | 95% CI |
| Rape myth acceptance | .125 | .046 | 2.72 | .007 | [.035, .215] |
| Sexual harassment perpetration | .275 | .094 | 2.92 | .004 | [.090, .460] |
| Age | -.001 | .007 | -.193 | .847 | [-.016, .013] |
| Religiosity | .030 | .019 | 1.57 | .118 | [-.008, .067] |
| Political orientation | .012 | .017 | .697 | .486 | [-.022, .046] |
| Gender | -.090 | .049 | -1.82 | .070 | [-.187, .007] |
| Confrontation type | .110 | .033 | 3.33 | < .001 | [.045, .175] |
| *Note*. Given the small proportion of non-binary participants, only men and women were included for these analyses. Racial identity was not included in the model as both samples have limited racial diversity. Women were the reference group for the Gender predictor variable and the Guilty confrontation condition was the reference group for the confrontation type predictor variable. | | | | | |

| **Table D**.  *Regression Coefficients for a Multivariate Multiple Regression Analysis Predicting Deny, Attack, and Reverse Subscales from Rape Myth Acceptance and Sexual Harassment Perpetration in the Community Sample (N = 333).* | | | | | |
| --- | --- | --- | --- | --- | --- |
|  | Deny | | | | |
| **Predictor variables** | *B* | *SE* | *t* | *p* | 95% CI |
| Rape myth acceptance | .060 | .046 | 1.32 | .188 | [-.029, .150] |
| Sexual harassment perpetration | .508 | .095 | 5.34 | < .001 | [.321, .695] |
| Age | -.008 | .004 | -2.09 | .037 | [-.016, -.001] |
| Religiosity | .074 | .049 | 1.50 | .134 | [-.023, .170] |
| Political orientation | .013 | .038 | .341 | .733 | [-.061, .087] |
| Gender | .096 | .129 | .748 | .455 | [-.157, .349] |
| Confrontation type | .687 | .135 | 5.08 | < .001 | [.421, .953] |
|  | Attack | | | | |
|  | *B* | *SE* | *t* | *p* | 95% CI |
| Rape myth acceptance | .130 | .039 | 3.37 | < .001 | [.054, .207] |
| Sexual harassment perpetration | .669 | .081 | 8.29 | < .001 | [.510, .828] |
| Age | -.002 | .003 | -.490 | .624 | [-.008, .005] |
| Religiosity | .179 | .042 | 4.30 | < .001 | [.097, .262] |
| Political orientation | .037 | .032 | 1.16 | .246 | [-.026, .100] |
| Gender | -.062 | .109 | -.565 | .572 | [-.277, .153] |
| Confrontation type | .079 | .115 | .684 | .494 | [-.147, .305] |
|  | Reverse | | | | |
|  | *B* | *SE* | *t* | *p* | 95% CI |
| Rape myth acceptance | .144 | .041 | 3.49 | < .001 | [.063, .225] |
| Sexual harassment perpetration | .588 | .086 | 6.83 | < .001 | [.419, .757] |
| Age | .002 | .004 | .415 | .678 | [-.006, .009] |
| Religiosity | .147 | .045 | 3.31 | .001 | [.060, .235] |
| Political orientation | -.003 | .034 | -.075 | .940 | [-.070, .065] |
| Gender | -.063 | .117 | -.536 | .592 | [-.292, .167] |
| Confrontation type | .040 | .123 | .326 | .744 | [-.201, .281] |
| *Note*. Given the small proportion of non-binary participants, only men and women were included for these analyses. Racial identity was not included in the model as both samples have limited racial diversity. Women were the reference group for the Gender predictor variable and the Guilty confrontation condition was the reference group for the confrontation type predictor variable. | | | | | |

After including the control variables in the model, the interpretation of the results for the undergraduate sample remains the same: rape myth acceptance and sexual harassment perpetration predict Attack and Reverse responses, but not Deny responses. Adding the control variables to the model for the community sample rendered RMA as a non-significant predictor of Deny responses (this is different from the model without the control variables, which finds RMA to predict Deny responses). However, interpretation for the remaining variables is the same: sexual harassment perpetration predicts Deny, Attack, and Reverse responses, and RMA predicts Attack and Reverse responses.

Provided that the results are very similar and that we have no theoretical basis for controlling for the demographic variables (and controlling for the confrontation type had no substantial effect on results, as described above), we prefer to retain the simpler model for our paper.

*In the discussion the authors explain that, because this is a cross-sectional correlational study, the order of the variables does not matter. I disagree. Ultimately, the framework of the study suggests understanding RMA and DARVO is important for identifying risk factors for sexual harassment. So, sexual harassment is the outcome of interest, and the authors are interested in assessing to what extent RMA and DARVO—two attitude related factors—predict sexual harassment—a behavior. For this reason, I believe that DARVO and RMA should be entered into models as predictors of sexual harassment, along with controls (e.g., demographics, confrontation type, sample type, etc.). Doing it this way might reveal that RMA predicts sexual harassment perpetration but DARVO does not, or that RMA mediates the relationship between DARVO and sexual harassment, as the theory might suggest.*
Thank you for this thoughtful suggestion regarding our conceptual framework and analyses.

We may have unintentionally misrepresented the framework of the study, as we considered both sexual harassment and rape myth acceptance to be equally important when considering associations with DARVO – although we believe demonstrating a link between DARVO use and sexually harassing behaviors is meaningful, we do not intend to focus on sexual harassment as the primary outcome variable. In our view, DARVO is a potential behavioral mechanism that promotes or facilitates both rape myth acceptance and sexual harassment.

With this said, we computed a model predicting sexual harassment perpetration from DARVO and RMA while controlling for demographic variables (gender, age, religiosity, and political beliefs) and confrontation type. Using a General Linear Model, we identified the following results as shown in Table E below:

| **Table E**  *General Linear Model Predicting Respondents’ Sexual Harassment Perpetration from DARVO and RMA* | | | | | |
| --- | --- | --- | --- | --- | --- |
| Predictor | B | SE |  | *t* | *p* |
| Community Sample | | | | | |
| DARVO | .297 | .035 |  | 8.48 | <.001 |
| Rape myth acceptance | .187 | .022 |  | 8.41 | < .001 |
| Religiosity | -.031 | .027 |  | -1.15 | .251 |
| Political orientation | .008 | .020 |  | .405 | .686 |
| Age | -.001 | .002 |  | -.382 | .703 |
| Gender | .060 | .068 |  | .878 | .380 |
| Confrontation type | -.014 | .072 |  | -.192 | .848 |
| Undergraduate Sample | | | | | |
| DARVO | .064 | .024 |  | 2.69 | .007 |
| Rape myth acceptance | .101 | .020 |  | 4.99 | < .001 |
| Religiosity | -.020 | .009 |  | -.2.29 | .022 |
| Political orientation | .000 | .008 |  | -.020 | .984 |
| Age | .010 | .003 |  | 2.96 | .003 |
| Gender | .107 | .022 |  | 4.91 | < .001 |
| Confrontation type | .009 | .015 |  | .618 | .537 |
| *Note*. Given the small proportion of non-binary participants, only men and women were included for these analyses. Racial identity was not included in the model as both samples have limited racial diversity. Women were the reference group for the Gender predictor variable and the Guilty confrontation condition was the reference group for the confrontation type predictor variable. | | | | | |

In both samples, individuals’ DARVO use and rape myth acceptance are statistically significant predictors of sexual harassment after controlling for demographic variables and confrontation type. While we are not interested in predicting sexual harassment alone, these results offer preliminary evidence that DARVO explains unique variance in sexual harassment perpetration above and beyond rape myth acceptance. However, as stated above, we do not want to give precedence to sexual harassment perpetration in the current study. We prefer to maintain both rape myth acceptance and sexual harassment perpetration as predictors of individuals’ DARVO use.

*Study 1/Study 2 separation

It was unclear why the authors separated this into two studies. I understand that the source of data was different, and accessing the two different samples followed different procedures, but all other aspects of the study appeared to be the same. So, why not simply describe one study, include both data collection procedures, and then describe one set of results. You could always do a split sample analysis splitting the sample of students and MTurk respondents so that you still get to replicate findings in two groups. This just does not seem like two studies to me. It is one study, with two different samples.*

We agree that the manuscript would be more coherent if presented as a single study with two samples. We have therefore revised the manuscript to describe one study that contains two distinct samples; specifically, we revised the method, results, and discussion sections to reflect this. For clarity, we have kept statistical analyses separated by sample.

*The large decrease in the MTurk sample size, after removing problematic responses is a problem. Almost half the sample was dropped. Can the authors clarify if the dropped respondents were substantively different from the final sample?*

We agree that the number of problematic responses in the MTurk data is concerning – we did not anticipate needing to screen out this number of responses from the data. As described in the participants section, part of our data screening process involved examining written responses to an open-ended question asking participants to describe the confrontation they elected to report on for the survey. Participants who provided incoherent responses to this open-ended question were dropped from analyses. For example, some participants (or bots, as we suspect) wrote, “Voluntary False Confessions. Kassin and Wrightsman33 initially defined a voluntary false confession as one that is offered in the absence of police interrogation,” “specific places,” and “The researchers were inspired to investigate the link between anger and guilt five years ago after discussing true crime documentaries and the dynamics of the falsely accused being interviewed by police.” These kinds of responses suggested the individuals’ data would not be acceptable for analyses.

We tested for differences in study variables (DARVO, rape myth acceptance, and sexual harassment perpetration) between the retained data and the problematic dropped data. Table F below contains the means, standard deviations, and independent-samples *t*-tests for these comparisons. All means for the dropped data group are higher.

| **Table F**  *Independent-Samples t-Tests Comparing Retained and Dropped Data for the MTurk Sample* | | | |
| --- | --- | --- | --- |
|  | Retained data  *M* (*SD*) | Dropped data  *M* (*SD*) | Independent-samples *t*-test |
| DARVO | 1.88 (.87) | 3.10 (.77) | *t*(580) = -17.15, *p* < .001 |
| Rape myth acceptance | 2.83 (1.48) | 4.84 (1.18) | *t*(580) = -17.18, *p* < .001 |
| Sexual harassment | 1.60 (.65) | 2.77 (.62) | *t*(578) = -21.33, *p* < .001 |

Our findings would appear to be slightly more compelling if we were to use the dropped data for analyses. For example, the correlation between DARVO and rape myth acceptance with the retained data is .597, as reported in the manuscript; including the dropped data increases this correlation to .727. The unstandardized beta values for the multivariate test also appear to be larger when the dropped responses are included. Importantly, however, the inclusion (or exclusion) of the problematic data does not change the basic interpretation of the results (i.e., no results are rendered insignificant when they were previously significant and vice versa).

In striving to present high quality data, we prefer the problematic data remain omitted from analyses, even if it comes at a small cost to the ostensible magnitude of some of our findings.

*Discussion

The authors suggest that differences in demographics could account for differences in effect sizes/correlations in the Mturk and student samples. This statement could be better supported by including demographics as controls in the multivariate models.*

We did speculate that differences in demographic variables might have contributed to the observed differences between the samples. And, after conducting analyses controlling for the demographic variables in the study, some potentially interesting but ultimately inconsistent findings emerged regarding the predictive power of the demographic variables. We believe that additional research that intentionally explores the role of demographic variables might offer clearer insights than the current study. We have updated the section suggesting that demographic variables might be contribute to the observed differences to list two specific demographic variables (i.e., education level and socioeconomic status) that we think may have an effect on study variables but were not evaluated in the current study:

“This difference, while notable, is not central to our hypotheses; however, it suggests differences in some demographic variables may attenuate the strength of the associations between DARVO, rape myth acceptance, and sexual harassment perpetration. Additional DARVO research that focuses specifically on the role of education level, socioeconomic status, and other demographic variables would be better equipped to explore this issue.”

*The debate of which came first—the behavior or the attitude—is ongoing. Still, when it comes to rape myths, the research tends to frame the attitude as happening first. People who are high in RMA are more likely to perpetrate sexual violence, more likely to blame victims, less likely to acknowledge or report their own rape. So, the comment on pg 26, line 524-25 is backwards. RMA/rape culture may contribute to DARVO, rather than DARVO somehow increasing rape myth acceptance.*

The sentence in question (lines 524-525, page 26), we believe, is: “These findings suggest

that DARVO use indicates greater support for and participation in rape culture.” This appears to us as a non-directional statement; it only intends to say that people who use DARVO are also more likely to support and participate in rape culture. If this comment actually refers to another sentence in the manuscript, we are happy to revisit it.

*Limitations

If the “confrontation” was one that the respondent recalled, rather than a hypothetical scenario, then this this a limitation that should be noted. Depending on when the even occurred, respondents could forget their response to confrontation. So there needs to be more clarity on what the confrontation component is, and how it was assessed, and if memory may be an issue.*
We have added the following to the limitations section:

“The current study is the first to evaluate individuals’ self-reported DARVO use. However, as with most self-report data on past experiences, respondents’ capacity to accurately recall and report the event may vary – some respondents may have a clear and accurate recall of the confrontation they were asked to report on, while other respondents’ recollections (and therefore reporting) may be less precise. This issue could be potentially addressed by asking respondents to recall a confrontational experience that occurred within a recent timeframe, such as within that past 6 months.”

We also added greater clarity to how respondents were asked to report on their DARVO use. In the materials section, we state:

“The scale did not impose limitations with respect to when the confrontation occurred or the specific nature of the confrontation – respondents could report on a confrontation that happened at any time and, as long as it was about the worst or most serious thing they were accused of doing, could be about a range of behaviors.”

*Typos and grammar issues

P. 4, line 82, 85~~;~~ p 7 line 164; p 9, line 196; p. 12, line 273; p. 25, line 519;*

Thank you for bringing these typos to our attention. We have addressed each one in the revised manuscript.
