# Supplementary material for: Associations between defensive victim-blaming responses (DARVO), rape myth acceptance, and sexual harassment
Source: PLoS One. 2024 Dec 4;19(12):e0313642. doi: 10.1371/journal.pone.0313642 (PMC11616802; doi:10.1371/journal.pone.0313642)
Supplement: Attachment — Submitted filename: PLOS ONE_DARVO_2nd Review.docx [file pone.0313642.s002.docx]

PLOS ONE Article Review—Response to revision

I see the authors completed extensive revisions of the manuscript. I believe their hard work has paid off and the study is much improved! Below I have left a few additional comments/ recommendations that I hope could further improve the manuscript.

I recommend a paragraph at the end of the introduction, before the section on RMA that briefly, in a few sentences, states the research questions an how the authors’ study addresses those questions. As of now, there is no statement of what the study asks, addresses, or does (except for the abstract), until the current study section on page 8. As the reader, it is difficult to follow the reviewed literature, without getting a preview of why it matters. A brief paragraph noting this in the introduction would allow the reader to know what to look for and will prepare them to identify the relationships that the authors set up in the lit review.

DARVO is the clear focus of the study, and is frequently referenced first, such as in the methods section talking about measures. Logically, and for symmetry, it might make more sense to put the DARVO section first in the literature review as well, followed by the section on RMA.

On page 6, the authors comment on the experiences of their participants as evidence that DARVO can be used in many different contexts. Please do not use data/findings from the current study in the literature review justifying the study. Perhaps there is other research that reveals this, that the authors can draw on. Otherwise, they might note the types of incidents that DARVO has been documented in, and then speculate that it may also be used in a wider range. It just seems off to use findings from the study to justify or frame the study (that in the narrative has not been done yet).

On page 8, the authors state the purpose of the study (lines184-186): “The purpose of the current study was to evaluate individuals’ own general use of DARVO as a response to confrontations regarding a range of wrongdoing, including possible incidents of sexual violence as well as a variety of other incidents, and their rape myth acceptance.” This makes it sound like they were interested in exploring the variety of confrontations or accusations that the accused may use DARVO in, such as using a content analysis to categorize characteristics of the confrontation (severity of accusation, relationship between accuser and accused) and see if DARVO use varies by these characteristics. The next statement seems to more accurately reflect the actual purpose of the study and what the authors did: “To further explore DARVO’s connection to sexual violence, the current study also sought to investigate the general use of this tactic in conjunction with a behavioral measure of engagement in sexual harassment.” The second statement is reiterated further, with: “The current study aimed to test associations between DARVO use, rape myths, and sexual harassment perpetration among two different samples: undergraduates and community members recruited online.” These later two statements are clearer and more specific than the first, which is misleading. If a clear statement of what the authors did/the purpose (similar to these second two statements) was included in the introduction, the first statement of purpose presented here may make more sense.

While the authors note that people described a wide range of confrontations, given the theoretical and empirical link of DARVO to sexual violence, it would be helpful to know the proportion of respondents who described a confrontation about a sexual violence related incident.

In the methods section, page 15, please clarify if people were randomly assigned to the confrontation conditions, or otherwise clarify how this assignment was determined.

Research studies show that completing an RMA scale before reviewing a vignette and responding about victim blame or rape proclivity can prime respondents to respond more negatively. Can the authors describe the order of measures in the survey? Did respondents review the DARVO section first, followed by RMA, and then the sexual harassment bit? Was order randomized to prevent priming effects?

Tables 2 and 3 show tests comparing DARVO, RMA, and sexual harassment scores for men and women but the authors never noted possible gender differences as one of their research questions or predicted a relationship among their hypotheses. If this was an area of interest at the outset, please make it clear and include the hypothesis. If this was exploratory, please make that clear why this was explored, and not predicted. Given the number of tests conducted, including those that were not hypothesized, the authors may want to consider using a more stringent alpha for the cutoff.

What was the pre-determined alpha value? I presumed, given it was not stated (or did I miss this?), that it was .05. But on page 25, line 502, they note the p-value for the relationship between RMA and Reverse as .071. Perhaps this is a typo, as the same effect in the table is listed with a p = .017? Please clarify and double-check all your numbers.

The stronger relationship between RMA and DARVO among the community sample could be due, in part, to the higher endorsement of RMA among this group. If I am reading the tables correctly, Mturk-worker RMA was almost twice that of the students, and both were below the neutral point. So, in both cases, the relationship with DARVO could be driven primarily by the relatively few individuals at the higher end of the scale, with scores above the neutral point. It may also be related to age. I am not surprised that none of the demographic characteristics were significant in the model after including RMA, as many studies report that RMA mediates the relationship between demographic characteristics like age, gender, and race and the outcomes of interest, typically victim blame or rape proclivity. But, age is a really good predictor of RMA, and older people tend to have higher RMA than younger people. In unpublished work of my own, I have also found that somewhat different demographic characteristics predict RMA/victim blame among students versus MTurk workers, so I can confirm informally that unmeasured demographic characteristics may matter.

Line 614—the authors write that some underlying factor may lead people to engage in the three variables.” Perhaps “phenomena” instead of “variables” is more precise.

Line 638 refers to “Study 2.”

Typos in lines 550, 555, 580, 603, 653.
