# Supplementary material for: Associations between defensive victim-blaming responses (DARVO), rape myth acceptance, and sexual harassment
Source: PLoS One. 2024 Dec 4;19(12):e0313642. doi: 10.1371/journal.pone.0313642 (PMC11616802; doi:10.1371/journal.pone.0313642)
Supplement: Attachment — Submitted filename: PONE-D-23-33892R1_Response to Reviewers_9 Oct 2024.docx [file pone.0313642.s003.docx]

Response to Reviewers

PONE-D-23-33892R1

We greatly appreciate the reviewer's thoughtful and thorough comments on the manuscript. We have made additional revisions based on these comments – we believe these changes have further strengthened the manuscript. The reviewer’s comments and our responses appear below.

I see the authors completed extensive revisions of the manuscript. I believe their hard work has paid off and the study is much improved! Below I have left a few additional comments/ recommendations that I hope could further improve the manuscript.

I recommend a paragraph at the end of the introduction, before the section on RMA that briefly, in a few sentences, states the research questions an how the authors’ study addresses those questions. As of now, there is no statement of what the study asks, addresses, or does (except for the abstract), until the current study section on page 8. As the reader, it is difficult to follow the reviewed literature, without getting a preview of why it matters. A brief paragraph noting this in the introduction would allow the reader to know what to look for and will prepare them to identify the relationships that the authors set up in the lit review.

We have added the following paragraph that appears at the end of the introduction section and before the literature review:

“The current study seeks to quantitatively explore an understudied factor that may aid in the perpetration of sexual violence: DARVO (Deny, Attack, Reverse Victim and Offender). DARVO is a defensive behavior characterized by victim blaming and deflecting responsibility for wrongdoing. By intentionally distorting narratives of sexual violence in their favor, perpetrators may increase their chances of avoiding consequences for committing sexually violent acts. We therefore aimed to test for an association between individuals’ use of DARVO and their engagement in sexual harassment, a common form of sexual violence. Moreover, as myths about rape reflect victim-blaming attitudes, we also examined DARVO use and acceptance of rape myths. We predicted that DARVO use would be positively and significantly associated with sexual harassment perpetration and rape myth acceptance in community and undergraduate samples.”

DARVO is the clear focus of the study, and is frequently referenced first, such as in the methods section talking about measures. Logically, and for symmetry, it might make more sense to put the DARVO section first in the literature review as well, followed by the section on RMA.

We have reorganized the literature review so that the DARVO section appears first. Discussion of rape myths within the DARVO section has been moved to the Rape Myths section of the literature review.

On page 6, the authors comment on the experiences of their participants as evidence that DARVO can be used in many different contexts. Please do not use data/findings from the current study in the literature review justifying the study. Perhaps there is other research that reveals this, that the authors can draw on. Otherwise, they might note the types of incidents that DARVO has been documented in, and then speculate that it may also be used in a wider range. It just seems off to use findings from the study to justify or frame the study (that in the narrative has not been done yet).

The literature review does not discuss findings from the current study. This is the section in question:

“A study examining the prevalence of DARVO asked 138 undergraduates to share their experiences confronting someone else over a wrongdoing [28]. Nearly 72% of the sample reported the person they confronted had used phrases representing all three elements of DARVO – denials, attacks, and reversals – during the confrontation. The wrongdoings reported by the participants in this study encompassed a variety of interpersonal mistreatment, which ranged from social transgressions (e.g., having a secret betrayed by a family member or close friend) to more serious cases of abuse (e.g., sexual assault and child abuse), suggesting DARVO is used in contexts both in and outside of sexual violence.”

“The wrongdoings reported by the participants in this study...” refers to the study introduced in the previous lines (previously reference 28). To add clarity, however, we have revised this phrase to now read, “Nearly 72% of the sample reported the person they confronted had used phrases representing all three elements of DARVO – denials, attacks, and reversals – during the confrontation. The wrongdoings reported by the sample encompassed a variety of interpersonal mistreatment...” A citation for the study discussed now appears at the end of this sentence as well.

On page 8, the authors state the purpose of the study (lines184-186): “The purpose of the current study was to evaluate individuals’ own general use of DARVO as a response to confrontations regarding a range of wrongdoing, including possible incidents of sexual violence as well as a variety of other incidents, and their rape myth acceptance.” This makes it sound like they were interested in exploring the variety of confrontations or accusations that the accused may use DARVO in, such as using a content analysis to categorize characteristics of the confrontation (severity of accusation, relationship between accuser and accused) and see if DARVO use varies by these characteristics. The next statement seems to more accurately reflect the actual purpose of the study and what the authors did: “To further explore DARVO’s connection to sexual violence, the current study also sought to investigate the general use of this tactic in conjunction with a behavioral measure of engagement in sexual harassment.” The second statement is reiterated further, with: “The current study aimed to test associations between DARVO use, rape myths, and sexual harassment perpetration among two different samples: undergraduates and community members recruited online.” These later two statements are clearer and more specific than the first, which is misleading. If a clear statement of what the authors did/the purpose (similar to these second two statements) was included in the introduction, the first statement of purpose presented here may make more sense.

We have removed the misleading statement about the study’s purpose on page 8 and have replaced it with the following: “The purpose of the current study was to explore DARVO’s connection to sexual violence by investigating the general use of this tactic in conjunction a behavioral measure of engagement in sexual harassment.”

Moreover, the newly added paragraph at the end of the introduction includes the following statement, which we hopes adds further clarity: “We therefore aimed to test for an association between individuals’ use of DARVO and their engagement in sexual harassment, a common form of sexual violence. Moreover, as myths about rape reflect victim-blaming attitudes, we also examined DARVO use and acceptance of rape myths. We predicted that DARVO use would be positively and significantly associated with sexual harassment perpetration and rape myth acceptance in community and undergraduate samples.”

While the authors note that people described a wide range of confrontations, given the theoretical and empirical link of DARVO to sexual violence, it would be helpful to know the proportion of respondents who described a confrontation about a sexual violence related incident.

We conducted a rudimentary examination of participants’ written responses describing the nature of the confrontation they experienced. Across both student and MTurk samples, 16 total participants elected to report on a time they were accused of sexual misconduct. In the student sample, 3 descriptions of sexual misconduct accusations were in the Guilty condition, and 7 were in the Innocent (i.e., “falsely accused”) condition. Among MTurk participants, 2 described being accused of sexual misconduct in the Guilty condition and 4 described being falsely accused of engaging in sexual misconduct (Innocent condition).

The following information has been added to the descriptive findings section of the results for the undergraduate and community samples, respectively: “Among the undergraduates who provided written details of the confrontation they experienced, 10 individuals described being accused of sexual misconduct,” and “An analysis of participants’ written descriptions of the confrontation revealed that 6 respondents had been confronted about an issue relating to sexual misconduct.”

In the methods section, page 15, please clarify if people were randomly assigned to the confrontation conditions, or otherwise clarify how this assignment was determined.

In the materials subsection on page 15, the manuscript originally stated: “In the current study, which uses data originally collected for multiple projects, participants were randomly assigned to report their DARVO use in one of two contexts....” To add more information about the random assignment procedure used in the study, we have revised the wording so that it now reads as: “In the current study, which uses data originally collected for multiple projects, participants were randomly assigned using the randomizer function in Qualtrics to report their DARVO use in one of two contexts....”

Research studies show that completing an RMA scale before reviewing a vignette and responding about victim blame or rape proclivity can prime respondents to respond more negatively. Can the authors describe the order of measures in the survey? Did respondents review the DARVO section first, followed by RMA, and then the sexual harassment bit? Was order randomized to prevent priming effects?

As noted in the procedure section, " All participants were presented with the study measures in the same order (i.e., demographic questions, DARVO-USE, rape myth acceptance, and sexual harassment perpetration).” As RMA appeared after participants reported on their previous experiences using DARVO, there would be no priming effect on the DARVO-USE measure. Sexual harassment perpetration appeared after RMA, but this was a behavioral scale and not a judgement- or proclivity-based measure.

Tables 2 and 3 show tests comparing DARVO, RMA, and sexual harassment scores for men and women but the authors never noted possible gender differences as one of their research questions or predicted a relationship among their hypotheses. If this was an area of interest at the outset, please make it clear and include the hypothesis. If this was exploratory, please make that clear why this was explored, and not predicted. Given the number of tests conducted, including those that were not hypothesized, the authors may want to consider using a more stringent alpha for the cutoff.

We included comparisons of men and women’s mean scores – even if they were not hypothesized – as a standard practice of descriptive reporting. We believe gender differences are generally useful to present for these variables for researchers who may wish to conduct meta-analyses. For instance, in the literature review, we noted previous systematic research has revealed consistent gender differences in rape myth acceptance (i.e., “In a meta-analysis of 37 studies, researchers identified that men report greater rape myth acceptance than women [23]”). However, we have added the following to the Current Study section: “Moreover, given gendered differences in RMA [23] and sexual harassment perpetration [34], we expected to observe higher scores among men than women for these variables. Gender differences in DARVO use were explored without predictions.”

We recognize some researchers may use an adjusted alpha value for comparisons between groups on multiple dependent variables, like those in Tables 2 and 3. García-Perez (2023) suggests that adjusting alpha levels is justified in cases where many individual tests are used to evaluate a single omnibus null (i.e., the omnibus null is rejected if one of the individual tests is statistically significant). This particular scenario does not appear in the current study; consequently, we did not use alpha adjustments. Moreover, when applicable, we reported effect sizes and confidence intervals, which contribute additional information about the analyses beyond statistical significance.

What was the pre-determined alpha value? I presumed, given it was not stated (or did I miss this?), that it was .05. But on page 25, line 502, they note the p-value for the relationship between RMA and Reverse as .071. Perhaps this is a typo, as the same effect in the table is listed with a p = .017? Please clarify and double-check all your numbers.

The correct p-value for the association between RMA and Reverse is .017. This was accurately reported in Table 6 but mistyped in the text. We have corrected this typo in the manuscript and reviewed the statistical results for other errors.

The stronger relationship between RMA and DARVO among the community sample could be due, in part, to the higher endorsement of RMA among this group. If I am reading the tables correctly, Mturk-worker RMA was almost twice that of the students, and both were below the neutral point. So, in both cases, the relationship with DARVO could be driven primarily by the relatively few individuals at the higher end of the scale, with scores above the neutral point. It may also be related to age. I am not surprised that none of the demographic characteristics were significant in the model after including RMA, as many studies report that RMA mediates the relationship between demographic characteristics like age, gender, and race and the outcomes of interest, typically victim blame or rape proclivity. But, age is a really good predictor of RMA, and older people tend to have higher RMA than younger people. In unpublished work of my own, I have also found that somewhat different demographic characteristics predict RMA/victim blame among students versus MTurk workers, so I can confirm informally that unmeasured demographic characteristics may matter.

We appreciate this comment about the differences in RMA between the community and student samples. We agree, as we note in the limitations and future directions, that future research would benefit from evaluating more demographic variables to more thoroughly examine RMA in this context.

Regarding outliers, we examined scatterplots and z-scores to identify high outliers in RMA scores. Although scatterplots revealed the presence of some elevated scores, none appeared to be visually extreme. In the undergraduate sample, we re-ran correlations between DARVO and RMA excluding individuals with RMA z-scores of 3 or higher (*n* = 10)*;* the correlation was smaller but still statistically significant (*r* = .094, *p* = .023). A manual inspection of these individuals’ data did not reveal any concerning patterns (e.g., straight-lining). The MTurk sample did not contain any RMA z-scores of 3 or higher.

Line 614—the authors write that some underlying factor may lead people to engage in the three variables.” Perhaps “phenomena” instead of “variables” is more precise.

Thank you for this suggestion; we have replaced “variables” with “phenomena” in this sentence.

Line 638 refers to “Study 2.”

We have removed the sentence that refers to Study 2.

Typos in lines 550, 555, 580, 603, 653.

Thank you for drawing our attention to these typos. We have addressed each error in the manuscript.
